# Functional Region-Based Design Rules for Stress-Aligned Multi-Axis Fused-Filament Fabrication

**DOI:** 10.3390/polym18192323

**Published:** 2026-09-23

**Authors:** Diego Armando Aguirre Guerrero, Georg Jacobs, Kathrin Boelsen, Yujing Feng, Sebastian Hacker, Nils Speetzen, Mark Witte

**Affiliations:** 1 Institute for Machine Elements and Systems Engineering, RWTH Aachen University, 52062 Aachen, Germany; georg.jacobs@imse.rwth-aachen.de (G.J.); kathrin.boelsen@imse.rwth-aachen.de (K.B.); yujing.feng@imse.rwth-aachen.de (Y.F.); sebastian.hacker@imse.rwth-aachen.de (S.H.); 2 Visual Computing Institute, RWTH Aachen University, 52074 Aachen, Germany; speetzen@informatik.rwth-aachen.de; 3 Institute of Mechanism Theory, Machine Dynamics and Robotics, RWTH Aachen University, 52062 Aachen, Germany; witte@igmr.rwth-aachen.de

**Keywords:** additive manufacturing, fused filament fabrication, multi-axis deposition strategies, functional regions

## Abstract

Multi-axis fused-filament fabrication (FFF) expands the design space of polymer components by enabling spatially oriented filament paths. Existing stress-aligned deposition methods can align paths with principal stress directions, but they provide limited guidance for selecting local deposition strategies in mechanically critical regions where loads are introduced, transmitted, redirected, supported, or constrained. This paper proposes a functional region-based method for deriving reusable local deposition-design rules. The method identifies the local structural context and governing load-transfer mechanism in a representative bolted bracket and abstracts these regions into compression, open-hole tensile, and bearing-response benchmarks. The compression benchmark is evaluated experimentally. Candidate deposition strategies for the open-hole tensile and bearing-response benchmarks undergo qualitative comparative screening using an FEM model with the Tsai–Wu failure-initiation index. Selected configurations are further evaluated through physical testing on multi-axis FFF specimens. The physical tests show that continuous load-transmitting paths are favorable in the investigated open-hole and bearing-response cases, whereas adding local offset reinforcement did not automatically improve the tested configurations. The resulting deposition-design rules were applied to a representative load-bearing bracket, which reached 3.89 kN and provided component-level transfer-feasibility evidence under the investigated conditions.

## 1. Introduction

### 1.1. Motivation and Problem Statement

Additive Manufacturing (AM) enables the fabrication of customized and geometrically complex components with high design freedom and reduced material waste [1,2,3]. Among polymer-based AM processes, Fused-Filament Fabrication (FFF) is increasingly investigated for structural applications because of its accessibility, low cost, and broad material availability [3,4]. However, FFF components typically exhibit lower strength across interlayer bonds than along deposited filaments [5]. This process-induced anisotropy limits their structural applications [1]. Consequently, stiffness, strength, and failure behavior depend on both the filament material and the arrangement of deposited filaments [1,5,6]. In this work, the term deposition strategy describes this arrangement, including filament direction, layer ordering, filament continuity, and local density distribution. The deposition strategy is therefore a central design variable for improving structural performance [6,7].

The manufacturing system determines the available deposition strategies. Conventional three-axis FFF uses planar layers, which restrict the spatial orientation of filament paths [8]. Multi-axis FFF enables deposition along curved layers and spatially oriented paths [8,9]. This allows filament directions to follow three-dimensional (3-D) geometry contours and local load paths more closely [8,10]. These capabilities have motivated research into stress-aligned deposition strategies for both three-axis [11] and multi-axis manufacturing contexts [12,13,14,15,16,17]. Such strategies use mechanical information, particularly principal stress directions, to guide filament orientation and improve structural performance [8,9,10,18,19].

Two challenges remain. First, several approaches use a single principal stress direction to guide filament paths [14,19,20]. This can be effective where one load path dominates. However, it may be insufficient where several principal stresses are mechanically relevant or stress directions vary rapidly [10]. Second, the selected mechanical information must be translated into continuous, well-spaced, and manufacturable filament paths [20,21]. Addressing these challenges requires an interpretation of the stress field in relation to the local geometry, boundary conditions, and physical interactions. These factors are referred here as the local structural context.

This local structural context is particularly relevant in mechanically critical regions where loads are introduced, transmitted, redirected, supported, or constrained. Examples include contact interfaces, cross-section transitions, and geometric discontinuities such as holes, notches, cut-outs, and voids. Contact pressure, friction, bearing, sliding, and changes in geometry or stiffness can produce multiaxial and spatially varying stress states [22]. In such regions, a single principal stress direction may not adequately describe the local load-transfer mechanism. The deposition strategy must therefore account for how loads pass through the region, as well as their local stress orientations.

A function-oriented perspective describes mechanically critical regions according to their role within the component and links that role to geometry and physical interactions [23]. This abstraction motivates the derivation of local deposition-design rules that consider stress information, local structural context, manufacturability, and filament continuity.

### 1.2. State of the Art

This section first reviews the workflow for designing, planning, and verifying stress-aligned FFF components. It then examines how existing methods use mechanical information to generate filament paths. The review identifies the additional guidance needed to select suitable local deposition strategies.

Based on the iterative workflow reported in [24], Figure 1 summarizes four stages. These are geometry generation [24], initial structural analysis using the finite element method (FEM) [14], deposition-strategy planning [19], and failure-oriented FEM simulation [24]. Together, these stages connect component geometry, mechanical analysis, filament-path generation, and structural verification.

Geometry generation defines the component topology and provides the geometric basis for the subsequent structural analysis. The initial FEM analysis calculates the Cauchy-stress tensor field under the specified boundary conditions and load cases. Principal stress magnitudes and directions are obtained from this field. This information provides the mechanical input for deposition-strategy planning [13,15,20,21,24].

Deposition-strategy planning uses the stress field to define filament arrangements and generate corresponding paths. Existing stress-aligned path-generation methods can be broadly classified as explicit or implicit [16]. Explicit methods trace ordered point sequences along local principal stress directions, for example by constructing streamlines [15,16,21]. These methods align paths directly with the selected stress directions. However, controlling path spacing and satisfying manufacturing constraints remain challenging. On complex 3-D surfaces, spacing must be controlled along the surface rather than in a plane [16]. Directly converting the point sequences into filament paths can also produce overlaps, sharp turns, and short segments. These features can reduce manufacturability and create weak points under loading [13,15].

Implicit methods use stress information to construct scalar fields [14,17,19] or phase fields [25]. Deposition paths are then extracted as iso-value lines, level sets, or field intersections [14,17]. These methods support smooth path generation, although the resulting paths generally approximate the selected principal stress directions. Selecting a mechanically suitable direction remains a separate design decision where several principal stresses are relevant or stress directions vary rapidly [20]. Such cases require additional criteria for interpreting the local stress state and selecting the deposition strategy.

Recent approaches incorporate manufacturing constraints into deposition-strategy planning, including path spacing, deposition-width limits, curvature control, and machine accessibility [20,24]. The approaches discussed here focus on continuous fiber-reinforced polymers, in which the deposited fibers define the local reinforcement directions. The method in [20] uses a direction-field representation that treats opposite orientations along the same fiber direction as mechanically equivalent. Combined with a periodic scalar field, this representation enables paths with nearly uniform spacing and improves path generation around mechanically critical regions. However, the equivalence of opposite orientations does not, by itself, determine which principal direction should guide deposition in a multiaxial stress state. Likewise, suitable spacing does not by itself establish path continuity through a critical region.

In addition to implicit methods, ref. [24] addresses manufacturability through a broader optimization process. It jointly optimizes component geometry, manufacturable layers, and path orientations for continuous fiber-reinforced polymer composites. Constraints on machine motion, layer curvature, path curvature, and layer thickness are included explicitly. These advances support the generation of manufacturable paths. The remaining design question is how local load-transfer mechanisms should guide strategy selection and the required continuity through the surrounding load-bearing material. This question is particularly relevant near contact interfaces, geometric transitions, and discontinuities.

After deposition-strategy planning, failure-oriented FEM simulation evaluates the planned filament arrangement. Deposition-path information is transferred to the structural model and combined with orthotropic material properties, and failure criteria such as Tsai–Wu or comparable criteria [24,26,27]. This simulation allows candidate strategies to be compared in terms of their predicted mechanical response. The resulting comparisons can inform subsequent design iterations. However, evaluating a specified strategy does not, by itself, provide reusable rules for selecting an initial strategy before detailed path generation.

The reviewed work therefore addresses stress-aligned path generation, manufacturing constraints, and structural verification. Three needs remain relevant to the present study. First, selecting a local deposition strategy requires an interpretation of mechanically critical stress states that extends beyond a single principal stress direction. Second, strategy selection must consider the continuity required by the local load-transfer mechanism, together with other manufacturing constraints. Third, comparisons between candidate strategies must be translated into guidance that can be reused for recurring structural contexts.

The research gap addressed here is therefore limited guidance for deriving reusable local deposition-design rules for recurring mechanically critical regions. Such rules should connect the local structural context to strategy selection and support the subsequent generation of continuous, well-spaced, and manufacturable filament paths.

### 1.3. Research Question and Contributions

This work aims to develop and evaluate a method for deriving reusable local deposition-design rules for multi-axis FFF. The rules are intended to guide the selection of local deposition strategies before detailed path generation. They are also intended to guide the implementation of those strategies as continuous, well-spaced, and manufacturable filament paths.

In this work, a functional region is a mechanically critical region associated with a recurring local structural context and load-transfer mechanism. It comprises the geometry through which loads are introduced, transferred, redirected, supported, or constrained. Where applicable, it includes a physical interaction area and the surrounding load-transmitting material. This definition draws on function-oriented mechanical design, which links functions to geometry and physical interactions [23,28]. It also draws on AM design approaches that consider product functions alongside geometric features [29].

The proposed method considers each functional region together with its local stress state, manufacturing constraints, and required filament continuity. Alternative filament arrangements are defined as candidate deposition strategies. Their mechanical responses are compared under representative local loading conditions, with numerical and experimental evaluation selected for each benchmark. These comparisons support rules for subsequent strategy selection.

The research question of this paper is:

RQ. *How can reusable local deposition-design rules be derived for recurring functional regions in multi-axis FFF to guide strategy selection and the generation of continuous, well-spaced, and manufacturable filament paths?*

Two assumptions guide the method. First, local stress states must be interpreted in relation to the load-transfer mechanisms within functional regions. Second, candidate strategies must be compared under representative conditions to assess their structural performance and manufacturability. These assumptions lead to the following hypotheses:

H1. *Interpreting local stress states in the context of functional regions identifies deposition requirements that are not captured by a single principal stress direction alone.*

H2. *Comparing candidate deposition strategies under representative load-transfer conditions enables the derivation of reusable local deposition-design rules. This comparison combines failure-oriented FEM simulation and physical testing with assessments of manufacturability and filament continuity.*

Together, these hypotheses structure the proposed method by linking functional regions, local stress states, manufacturability constraints, and filament continuity to the derivation of reusable local deposition-design rules. Their evaluation is limited to the materials, specimens, load cases, and component demonstration examined in this study.

## 2. Materials, Methods, and Results

This section describes the proposed method together with the numerical and experimental evidence used to derive local deposition-design rules for multi-axis FFF components. The method comprises five interconnected steps that link component-level analysis, simplified benchmark evaluation, and component-level verification. First, functional regions are identified in a representative load-bearing component by analyzing their local structural context, contact interfaces, and load-transfer mechanisms. Second, these regions are abstracted into simplified benchmark cases, and candidate deposition strategies are defined. Third, material characterization and the failure-oriented FEM simulation procedure establish the basis for numerical evaluation. Fourth, the candidate strategies are evaluated numerically and experimentally, with the scope of evaluation defined for each benchmark. The resulting evidence is used to derive local deposition-design rules. Fifth, these rules are applied to the corresponding functional regions of the representative component to define a component-level deposition strategy. The resulting mechanical response is evaluated through component-level testing.

### 2.1. Local Structural Context and Load-Transfer Mechanisms of the Representative Load-Bearing Component

The first step applies the definition of functional regions introduced in Section 1.3 to the representative load-bearing component. Their identification considers geometry together with the local structural context, contact interfaces, and load-transfer mechanisms for the selected load case. This distinction is required because geometrically similar regions, such as bolted holes, can experience different local mechanical states depending on the direction and magnitude of the applied load.

The representative component is a bracket with bolted connections, as shown in Figure 2. For transverse loading, two idealized load-transfer regimes are considered: friction-dominated clamping and bearing-dominated bolt–hole contact. These terms describe the predominant load-transfer mechanism. In the friction-dominated clamping state, the bolt clamps the parts together, allowing the applied shear load to be transferred by friction at the clamped interfaces. For a simplified model with one effective friction interface, friction can support the applied shear force provided that $F_{shear}\leq \mu F_{clamp}$, where $F_{shear}$ is the magnitude of the shear force to be transferred, $F_{clamp}$ is the effective normal clamping force at that interface, and *µ* is the coefficient of friction [22,30]. Relative sliding can occur when this limit is exceeded. If the sliding closes the bolt–hole clearance, the bolt shank contacts the hole boundary and introduces bearing load transfer. This contact is characterized by normal contact pressure at the hole boundary.

The transition from friction-dominated clamping to bearing-dominated contact is particularly relevant for printed polymer or composite-metal joints, because preload loss can reduce the effective clamping force over time. Such loss can result from seating, local plastic deformation, and creep. In thermoplastic joints, creep involves time-dependent deformation through the thickness of the clamped material, which can progressively reduce the bolt preload. The extent of this deformation can depend on temperature, stress level, and humidity [31,32].

For the load case considered in Figure 2, the three bolted-hole regions share the same basic geometry but experience different local mechanical conditions. Under the illustrated loading and support arrangement, the upper region experiences predominantly compressive contact loading along the bolt axis. The two lower regions transfer transverse loads and can experience different contact states. Without direct bolt–hole contact, each hole interrupts the surrounding tensile load path. If relative sliding closes the bolt–hole clearance, the bolt shank contacts the hole boundary. This contact enables load transfer through bearing.

These observations identify three local conditions for benchmark evaluation: compression-dominated contact, an interrupted tensile load path without direct bolt–hole contact, and bearing-dominated bolt–hole contact. Compressive loading can occur in both the friction-dominated and bearing-dominated load-transfer states.

### 2.2. Benchmark Abstraction and Candidate Deposition Strategies

The second step abstracts the functional regions identified in the representative component into simplified benchmark cases. These benchmark cases allow selected local mechanical conditions to be evaluated under controlled and comparable conditions. Each benchmark isolates a selected aspect of the local response described in Section 2.1.

The compression-related benchmark represents local compressive loading associated with bolt-head contact and support reactions (Figure 3). It is evaluated experimentally using two-layer orientations: 0°, with the layers aligned parallel to the compressive load direction, and 90°, with the layers oriented transverse to it. This comparison qualitatively examines the influence of layer orientation on the compressive response. Its findings inform deposition-design rules within the examined loading conditions and orientations.

The open-hole tensile benchmark examines how a hole interrupts a tensile load path without direct bolt–hole contact (Figure 4). It focuses on the stress concentration caused by this geometric discontinuity. The benchmark captures this local effect without reproducing all the contact and friction conditions of a clamped joint. Instead, the bearing-response benchmark examines how load is transferred through contact between the bolt shank and the hole boundary (Figure 4). This contact produces concentrated normal pressure at the loaded part of the hole surface.

The open-hole tensile and bearing-response benchmarks are evaluated using failure-based FEM simulations and physical testing. The same four candidate strategy types are considered for both benchmarks. This enables their structural performance to be compared under two distinct hole-related loading conditions. For the open-hole tensile and bearing-response benchmark cases, four candidate deposition strategies are considered, as illustrated in Figure 4. They represent four selected design decisions motivated by the research gap in Section 1.2: alignment with each of two principal directions, an intermediate filament orientation, and local offset reinforcement around the hole boundary.

- Strategy A: alignment with the maximum principal stress direction.
- Strategy B: alignment with the principal direction with the second-largest absolute magnitude.
- Strategy C: alignment at 45° relative to the maximum principal stress direction.
- Strategy D: alignment with the maximum principal stress direction combined with local offset reinforcement around the contact interfaces.

Strategies A and B compare the suitability of the two selected principal directions for deposition. This comparison is relevant because mechanically critical regions can contain multiaxial stress states, similar principal stress magnitudes, or rapidly changing stress directions. Strategy C examines the structural response obtained with an orientation at 45° to the selected principal direction. Strategy D evaluates the structural effect of local offset reinforcement, including whether its connection to the surrounding filament arrangement supports effective load transfer.

The output of this step is a set of benchmark-specific candidate deposition strategies and evaluation cases. The compression-related benchmark provides experimental evidence for the two selected layer orientations. The open-hole tensile and bearing-response benchmarks provide numerical comparisons of configurations combining Strategies A–D and experimental evidence for selected configurations. Together, these evaluations support the subsequent derivation of local deposition-design rules.

### 2.3. Material Characterization and Failure-Oriented FEM Benchmark Evaluation

The third step combines material characterization with failure-based FEM simulations to screen the candidate deposition strategies introduced in Section 2.2. The characterization provides selected direction-dependent elastic and strength parameters for the orthotropic material model. The simulations use this model and a Tsai–Wu failure-initiation index to compare the open-hole tensile and bearing-response configurations. The numerical results support qualitative comparative screening under the adopted model assumptions. They are not quantitatively validated predictions of failure initiation, damage evolution, or ultimate load.

Figure 5 shows the specimen geometries used for material characterization, benchmark implementation, and physical evaluation. The tensile specimens are used to characterize the direction-dependent tensile response of robot-printed PLA. The compression specimens serve to characterize the direction-dependent compressive response and to realize the compression-related benchmark. The open-hole tensile and bearing-response specimens realize the corresponding benchmark cases for an interrupted tensile-load path and bearing-dominated bolt–hole contact, respectively.

The specimens were fabricated from blue PolyTerra PLA (Polymaker, Changshu, China) filament with a nominal diameter of 1.75 mm. A custom multi-axis FFF system based on a KUKA KR 6 R900 SIXX robot (KUKA Deutschland GmbH, Augsburg, Germany) was used. The robot moved and rotated the build platform beneath a fixed 0.8 mm nozzle while maintaining a downhand deposition orientation. The nozzle diameter was selected to accommodate wider extrusion roads and relatively thick layers, with the aim of supporting efficient fabrication of relatively large functional components. The nominal deposition width was 0.8 mm, the layer height was 0.5 mm, and the nozzle temperature was approximately 200 °C. Deposition speed varied with robot motion and reached a maximum of 70 mm/s. The extrusion feed rate was adjusted according to robot velocity using a rate parameter and associated calibration factors. Cooling was provided by one fan directed toward the nozzle tip. Its position was constrained by the clearance required for nonplanar printing, which limited how closely it could be placed to the nozzle.

Mechanical testing was performed using a Zwick/Roell universal testing machine (ZwickRoell GmbH & Co. KG, Ulm, Germany) equipped with a 10 kN load cell. The tensile, compression, open-hole tensile, and bearing-response tests were conducted using the specimen geometries and procedures based on DIN EN ISO 527-4, ASTM D695, ASTM D5766/D5766M, and ASTM D5961/D5961M, respectively.

For the open-hole tensile and bearing-response benchmarks, all specimens have a nominal thickness of 4 mm. Seven different stacking configurations were defined, each consisting of eight printed layers with a layer height of 0.5 mm (Table 1). These configurations were used to compare selected combinations of strategy type, stacking sequence, and local offset reinforcement around the hole boundary. Each letter denotes the strategy assigned to one layer. The letters A, B, C, and D denote the candidate deposition strategies defined in Section 2.2. All configurations are evaluated numerically, while selected configurations are manufactured and tested physically, as described in Section 2.4.

The comparisons address different aspects of deposition-strategy design. For example, comparing AABBBBAA with ABABABAB examines the influence of stacking sequence while retaining four A layers and four B layers. Both filament orientation and local offset reinforcement change between AAAAAAAA and DACBBCAD. Differences in their response therefore reflect the combined configuration and cannot be attributed to reinforcement alone.

Numerical evaluation of these configurations requires material properties that describe the direction-dependent response of robot-printed PLA. Material characterization therefore establishes the measured elastic and strength inputs for the orthotropic material model. It also provides experimental evidence for interpreting the response of tensile and compression-related functional regions. The material designation PLA-Robot used in Table 2 and Table 3 refers to this robot-printed PLA material.

Figure 6a shows the tensile stress–strain curves for the 100% infill specimens together with representative fractured samples. The tensile response depended strongly on the orientation of the printed material relative to the loading direction. The 0° specimens reached a tensile strength of 25.6 MPa, whereas the 90° specimens reached 8.68 MPa. This corresponds to a reduction of approximately 66% when loading changed from the filament direction to the layer-normal direction. The elastic modulus decreased from 1910 MPa in the 0° configuration to 1230 MPa in the 90° configuration. The 0° specimens therefore exhibited higher stiffness, higher tensile strength, and a more gradual nonlinear response before fracture.

Figure 6b shows the compressive stress–strain curves and representative compressed specimens. The orientation dependence differed from that observed in tension. The compressive stress values adopted as strength inputs were 35.8 MPa for the 90° configuration and 21.5 MPa for the 0° configuration. The 90° specimens compacted progressively without a clearly localized macroscopic fracture. The 0° specimens showed internal mid-plane delamination, with deformation interpreted as buckling. Within the tested orientations and loading conditions, alignment along the filament direction was favorable in tension, whereas the layer-normal configuration sustained a higher compressive stress. These compression-test inputs are used as effective parameters and do not separately identify intrinsic material failure and specimen instability.

The material characterization therefore supports the later rule derivation by showing that local deposition-design rules should consider the principal stress direction together with the sign of the stress and the associated loading mode. For the subsequent FEM evaluation, measured elastic and strength parameters were used as model inputs. The resulting orthotropic elastic model and failure criterion provide a simplified representation for comparing deposition strategies. They do not reproduce the observed nonlinear deformation or delamination.

Table 2 and Table 3 summarize the elastic and strength parameters used in the orthotropic material model and in the Tsai–Wu failure criterion. The elastic modulus and tensile strengths in material directions 1 and 3 were obtained from the 0° lying and 90° standing specimens, respectively. Direction 1 follows the filament, and direction 3 is normal to the layers. The compression-test values of 21.5 and 35.8 MPa were used as compressive strength inputs for directions 1 and 3, respectively. The elastic modulus and tensile and compressive strengths in material direction 2 were set equal to the corresponding values in direction 3.

The parameters *ν*_12_ = 0.43, *ν*_13_ = 0.37, *G*_12_ = 919 MPa, *G*_13_ = 844 MPa, and *S*_12_ = 25.5 MPa were adopted from comparable PLA data reported in [37]. Data for *ν*_23_ and *G*_23_ were unavailable. Values of 0.40 and 900 MPa, respectively, were assigned as modeling assumptions. *S*_13_ and *S*_23_ were also assigned, with both set equal to *S*_12_ at 25.5 MPa. The same parameter set was applied to all deposition strategies to provide a consistent basis for comparison.

This consistent assignment does not establish that the ranking is insensitive to the assumed properties. Elastic parameters can affect the failure index indirectly through stress redistribution, whereas the strength parameters enter directly into the Tsai–Wu criterion. Uncertainty in the literature-derived and assigned parameters may therefore affect both the failure-index values and the ranking of the candidate strategies. The numerical results are interpreted as qualitative comparisons under the stated material assumptions. The model has not been quantitatively validated for failure initiation and does not predict damage evolution or ultimate load. Explanations of the physical causes of failure require support from experimental observations.

The elastic and strength parameters of PLA-Robot were then assigned to the benchmark models. Each printed structure was represented as an orthotropic solid whose local material orientation followed the planned filament path. This representation accounts for the directional response of the printed material. Figure 7 illustrates this assignment and the calculation of the Tsai–Wu failure index. The same material parameters were used for all deposition strategies to provide a consistent basis for comparison.

The planned filament path was mapped onto the finite element mesh. A local material coordinate system was then assigned to each element. In this coordinate system, direction 1 follows the filament path, direction 2 represents the transverse in-plane direction, and direction 3 represents the layer-normal direction. The material properties were defined in these local coordinates, so the response of each element depended on its assigned filament orientation.

In the local material coordinate system (1, 2, 3), the constitutive relation is written as:

(1) $$\{\varepsilon \}=\left[S\right]\{\sigma \},$$

The strain and stress vectors are defined as

(2) $$\{\varepsilon \}={\left\{\begin{array}{c} \varepsilon_{1}\\ \varepsilon_{2}\\ \varepsilon_{3}\\ \gamma_{23}\\ \gamma_{31}\\ \gamma_{12} \end{array}\right\}}^{T},\;\;\{\sigma \}={\left\{\begin{array}{c} \sigma_{1}\\ \sigma_{2}\\ \sigma_{3}\\ \tau_{23}\\ \tau_{31}\\ \tau_{12} \end{array}\right\}}^{T}$$

The compliance matrix $\left[S\right]$ is defined by the engineering constants $E_{1}$, $E_{2}$, $E_{3}$, Poisson ratios $\nu_{12}$, $\nu_{13}$, $\nu_{23}$, and shear moduli $G_{12}$, $G_{13}$, $G_{23}$. Since these properties are assigned in the local material coordinate system, the constitutive response of each element depends on the local filament orientation.

Failure initiation is assessed using the 3-D Tsai–Wu criterion. The criterion represents failure as a scalar function of stress components and strength parameters, and accounts for different tensile and compressive strengths. It is therefore suitable for comparing anisotropic material responses under multiaxial stress states [38].

The failure index $FI_{TW}$ is given by:

(3) $$FI_{TW}=F_{1}\sigma_{1}+F_{2}\sigma_{2}+F_{3}\sigma_{3}+F_{11}\sigma_{1}^{2}+F_{22}\sigma_{2}^{2}+F_{33}\sigma_{3}^{2}+2F_{12}\sigma_{1}\sigma_{2}\;+2F_{13}\sigma_{1}\sigma_{3}+2F_{23}\sigma_{2}\sigma_{3}+F_{44}\tau_{23}^{2}+F_{55}\tau_{13}^{2}+F_{66}\tau_{12}^{2}$$

Failure initiation is indicated when

(4) $$FI_{TW}\geq 1$$

The coefficients are obtained from the directional strength parameters:

(5) $$F_{1}=\frac{1}{X_{T}}-\frac{1}{X_{C}},\;\;F_{2}=\frac{1}{Y_{T}}-\frac{1}{Y_{C}},\;\;F_{3}=\frac{1}{Z_{T}}-\frac{1}{Z_{C}},\;F_{11}=\frac{1}{X_{T}X_{C}},\;\;F_{22}=\frac{1}{Y_{T}Y_{C}},\;\;F_{33}=\frac{1}{Z_{T}Z_{C}},\;F_{12}=-\frac{1}{2}\sqrt{F_{11}F_{22}},\;\;F_{13}=-\frac{1}{2}\sqrt{F_{11}F_{33}},\;\;F_{23}=-\frac{1}{2}\sqrt{F_{22}F_{33}},\;F_{44}=\frac{1}{S_{23}^{2}},\;\;F_{55}=\frac{1}{S_{13}^{2}},\;\;F_{66}=\frac{1}{S_{12}^{2}}.$$

Here, *X*, *Y*, and *Z* denote the tensile and compressive strengths in the filament, transverse, and through-thickness directions, respectively. $S_{12}$, $S_{13},$ and $S_{23}$ denote the shear strengths. The stress components used in Equation (3) are evaluated in the same local material coordinate system as the orthotropic constitutive response. Figure 7b summarizes this stress transformation and failure-index calculation.

The open-hole tensile and bearing-response benchmark models were implemented in Abaqus/CAE 2022 using quadratic tetrahedral C3D10 elements. A global mesh size of 0.5 mm was used, corresponding to the layer height and thereby allowing layer-wise geometric variations to be preserved. In the vicinity of the open hole, the mesh was locally refined to capture local stress concentrations more accurately. Mesh convergence was assessed for both benchmark models using local element sizes of 0.5, 0.4, 0.3, and 0.25 mm while retaining the global mesh size of 0.5 mm. The relevant failure-index metric varied by less than 1% over the investigated refinement range, indicating that the failure-index response had converged sufficiently for the comparative assessment. The finest investigated local element size of 0.25 mm was used in the final models to provide a detailed representation of the stress field around the hole.

The boundary conditions and load application are shown in Figure 8. In the tensile model, a tensile force of 900 N was applied. In the bearing-response model, a force of 360 N was applied to induce bearing loading. The pin was modeled as a rigid cylindrical body with fixed ends. Contact between the rigid pin and the hole surface was modeled using finite-sliding surface-to-surface contact. Hard contact was defined in the normal direction with separation after contact allowed, while frictionless behavior was assumed in the tangential direction. The analyses were performed in Abaqus/Standard using the Static, General procedure with geometric nonlinearity enabled.

Within each benchmark, geometry, loads, constraints, contact definitions, and output requests were retained across the stacking configurations. Element-wise material orientations represented the local filament directions of each configuration. The orthotropic response and Tsai–Wu failure-initiation index were evaluated in these local material coordinates. With this setup, the numerical model is used only for comparative screening of candidate deposition strategies. While the model evaluates failure initiation under a given load, it does not consider stiffness degradation, element deletion, crack propagation, or progressive damage. Consequently, it cannot predict damage evolution or ultimate failure load.

Candidate deposition strategies are compared using the spatial distribution of the Tsai–Wu failure index, the location of the critical region, and the global structural response of the benchmark model. The numerical evaluation is performed for the open-hole tensile and bearing-response benchmarks. The compression-related benchmark is not simulated as a failure-oriented FEM screening case, because it is evaluated experimentally as part of the direction-dependent material characterization.

Figure 9 compares the Tsai–Wu failure-index distributions for the open-hole tensile benchmark at 900 N. This prescribed screening load was below the experimental peak force of every physically tested configuration. It provides a common loading condition for comparing the candidate strategies. In the AAAAAAAA configuration, the maximum index is 0.762 at the hole boundary. In BBBBBBBB, the maximum is 2.703 at the hole boundary, and the elevated-index region is broader. The mixed and locally reinforced configurations also reach their maxima at the hole boundary, with maximum values between 1.829 and 2.192, as listed in Table 4. These contours directly support comparisons of maximum magnitude, critical location, and spatial distribution. They do not by themselves demonstrate filament continuity or prove that a path transition caused the critical response; such interpretations require the deposition-path and material-orientation information defined in Figure 4 and Figure 7.

At 900 N, the nominal gross-section stress is approximately 6.25 MPa for a specimen width of 36 mm and thickness of 4 mm. This value is lower than the longitudinal tensile strength of the robot-printed PLA characterized in Figure 6 and Table 2 and Table 3. However, the hole amplifies the local stresses, which are evaluated in the material coordinate system associated with each filament orientation. Table 4 summarizes the maximum failure index and critical location for each configuration.

A maximum index below 1 indicates that the criterion was not reached at the evaluated locations. A value of 1 or above indicates that the modeled initiation threshold was reached or exceeded locally. The simulation results show that AAAAAAAA is numerically most favorable (0.762), and BBBBBBBB is least favorable (2.703). All mixed and locally reinforced configurations have maxima above 1, but their suitability cannot be inferred from the maximum alone because the spatial distribution, load-transfer mechanism, path continuity, and manufacturability also matter. AAAAAAAA, ACACACAC, and DACBBCAD were therefore selected for physical testing to compare the favorable strategy.

Figure 10 compares the Tsai–Wu failure-index distributions for the bearing-response benchmark at 360 N. This prescribed screening load was below the experimental peak force of every physically tested bearing configuration. The load was introduced through contact between the rigid pin and the loaded side of the hole. This loading condition combines contact pressure with local compression, shear, and local tensile stresses that may contribute to splitting. For every evaluated stacking configuration, the maximum index reported in Table 5 occurs at the loaded hole boundary.

At the loaded hole boundary, AAAAAAAA has the lowest maximum index (0.815) and a localized elevated-index zone, whereas BBBBBBBB has the largest maximum (2.144). ACACACAC and DACBBCAD have maxima of 1.131 and 1.672, respectively. The remaining mixed or locally reinforced configurations have maxima between 1.838 and 1.913, as summarized in Table 5.

The same threshold interpretation applies to the bearing-response results. AAAAAAAA remains below 1 at the evaluated locations. All other configurations exceed 1 locally, with BBBBBBBB having the highest maximum. The numerical comparison describes the calculated response at the prescribed load. Its relation to the experimental response is examined in Section 2.4.

Accordingly, AAAAAAAA, ACACACAC, and DACBBCAD were selected for both physical hole-related benchmarks. AAAAAAAA provides a continuous reference with the lowest maximum failure index. ACACACAC represents an alternating A/C strategy incorporating the 45° orientation. DACBBCAD combines A/B/C layers with local offset reinforcement. This selection allows the reference to be compared with two alternatives that incorporate different design features. The tests examine whether the numerical ranking and critical-region trends are supported experimentally. They provide evidence for assessing these strategies under the investigated conditions.

### 2.4. Physical Benchmark Testing and Rule Derivation

Physical benchmark testing examines whether the trends identified by the failure-oriented FEM benchmark evaluation are also observed in printed specimens. Based on Section 2.3, AAAAAAAA, ACACACAC, and DACBBCAD were selected for the open-hole tensile and bearing-response benchmarks. These configurations compare continuous alignment with the selected principal stress direction, an alternating strategy incorporating the 45° orientation, and a combination of orientations with local offset reinforcement. Three specimens were evaluated for each configuration in each hole-related benchmark. The reported peak forces are summarized as arithmetic mean ± sample standard deviation. These small samples support descriptive comparisons under the investigated conditions.

Figure 11 shows the experimental open-hole tensile force–nominal-strain curves and representative fractured specimens. AAAAAAAA had the highest mean peak force, 2.395 ± 0.239 kN. DACBBCAD was intermediate at 1.948 ± 0.077 kN, and ACACACAC had the lowest mean at 1.697 ± 0.139 kN. The observed ranking was therefore AAAAAAAA > DACBBCAD > ACACACAC. The mean for AAAAAAAA was approximately 41% higher than that for ACACACAC and 23% higher than that for DACBBCAD. These percentages describe differences between the sample means and do not establish statistical significance.

The representative fractured specimens show failure in the notched region for AAAAAAAA, a more diagonal fracture path for ACACACAC, and damage around the hole and adjacent raster transition for DACBBCAD. These visible locations are consistent with configuration-dependent load transfer, but the pictures alone do not establish path-transition causality. Among the tested configurations, the numerical screening ranked AAAAAAAA (0.762) ahead of ACACACAC (1.829) and DACBBCAD (1.904), whereas the experiment ranked AAAAAAAA ahead of DACBBCAD and ACACACAC. The numerical model therefore identified the same best configuration and the same hole-boundary critical region, but it reversed the order of the two alternatives. This comparison does not quantitatively validate failure initiation or peak-force prediction.

Figure 12 shows the bearing-response curves and representative failed specimens. With three specimens per configuration, AAAAAAAA had the highest mean peak force, 1.368 ± 0.081 kN. ACACACAC was intermediate at 1.237 ± 0.058 kN, and DACBBCAD had the lowest mean at 0.972 ± 0.146 kN. The values are means ± sample standard deviations. The ranking of the sample means was AAAAAAAA > ACACACAC > DACBBCAD. DACBBCAD showed the largest variation in peak force between specimens. The repeat curves also differed in displacement at peak force. Slippage of the specimens within the machine grips was observed during testing and contributed to the recorded displacement. This provides an explanation for intervals of increasing tool separation with little increase in force. Consequently, the plotted displacement includes movement at the grips and cannot be interpreted solely as deformation of the specimen or elongation of the hole.

The representative failed specimens show damage concentrated around the loaded hole. Crushing, splitting, and separation are possible explanations of the observed damage, but their individual contributions were not quantified. For the tested configurations, the numerical screening ranked AAAAAAAA (0.815) ahead of ACACACAC (1.131) and DACBBCAD (1.672), reproducing the experimental ranking. The numerical model also identified the loaded hole boundary as the critical region. This agreement provides qualitative experimental support for the comparative screening trend, but the 360 N field comparison and the experimental peak forces are not a quantitative validation of final failure.

AAAAAAAA had the highest mean peak force in both tested hole-related benchmarks. The open-hole benchmark represents a tensile-load path interrupted by a hole, whereas the bearing benchmark represents load introduction through contact at the hole boundary. Both comparisons favor the tested continuous-path configuration. However, DACBBCAD changes both filament orientation and local offset reinforcement, so its response does not isolate the contribution of reinforcement alone. The results support checking that local reinforcement remains connected to the surrounding load-transmitting paths. They do not establish that offset reinforcement is generally detrimental.

The compression-related benchmark provides separate orientation-dependent evidence. The 0° specimens showed internal mid-plane delamination associated with buckling, while the 90° specimens compacted more progressively and reached a higher reported compressive strength. Together, the benchmarks indicate that local strategy selection should consider loading mode, contact state, and path continuity. Table 6 states the resulting deposition principles, their supporting evidence, and their application limits.

### 2.5. Component-Level Transfer and Mechanical Component Testing

The final step transfers the benchmark-derived deposition-design rules to the representative bracket. It examines whether these rules can guide a deposition strategy for several interacting functional regions within a more complex geometry. With one component tested per strategy, the evaluation provides descriptive evidence of transfer feasibility under the investigated conditions.

Figure 13 connects the local rule assignments in Table 6 to their implementation on the robotic manufacturing system. The six-axis robot positions and reorients the build platform and component beneath a stationary, downward-facing extrusion head. Relative motion between the component and nozzle generates the deposition paths while maintaining downhand deposition. Implementation requires coordinated path planning, robot motion, and extrusion delivery. The component’s position on the platform and the attainable platform orientations also constrain the implementation. The local deposition strategy combines two decisions: the orientation of the layer planes and the filament arrangement within those planes.

The upper bolted region is treated as a compression-related contact region because the load is introduced mainly along the bolt axis. The planar-layer baseline uses layers oriented parallel (0°) to the local compressive direction. The curved-layer strategy reorients the layers toward the transverse orientation (90°) favored in the compression benchmark. A fully transverse orientation could not be achieved in the present setup. The maximum manufacturing tilt was limited to 60° by the downhand-deposition requirement, robot motion constraints, and component placement on the platform. This manufacturing tilt limit is distinct from the target layer orientation relative to the compressive load.

Within each layer, Strategy A defines the filament arrangement according to the selected principal-stress direction. The open-hole tensile rule was used to select this strategy for the upper region. AAAAAAAA achieved the highest mean recorded peak force among the physically tested configurations in both hole-related benchmarks. Thus, the bearing-response benchmark supports the same strategy choice, although it represents a different load-transfer mechanism.

The two lower bolted regions also experience compressive contact loading along the bolt axes due to clamping. The compression-related rule therefore guides their local layer orientation, targeting layer planes transverse to this compressive direction (90°). The attainable orientation remains subject to the manufacturing constraints. Clamping compression can coexist with transverse load transfer through friction or bolt–hole bearing. The layer orientation and the filament arrangement must therefore address these conditions together.

The transverse load transfer at the lower connections is evaluated using the friction-dominated and bearing-dominated states defined in Section 2.1. In the idealized friction-dominated state, shear is transferred at the clamped interfaces without direct bolt–hole contact. Each hole then interrupts the surrounding tensile load path, making the open-hole tensile rule relevant.

As the applied load increases, the shear demand at the lower connections increases. Once this demand exceeds the frictional resistance generated by the bolt preload, relative sliding occurs. When the sliding closes the bolt–hole clearance, the bolt shank contacts the hole boundary and introduces bearing load transfer. The bearing-response rule is therefore applied around the loaded hole boundary to account for this contact state during continued loading. Strategy A defines the corresponding filament arrangement within the selected layer planes. The compression-related rule addresses the layer orientation under clamping, while the bearing-response rule addresses the filament arrangement around the loaded hole boundary.

Figure 14 shows the component-level test setup and the force–displacement responses of a planar-layer baseline and the rule-based curved-layer bracket. For the manufacturing and test record, the components used the same bracket geometry, PLA filament, robot-based manufacturing setup, and component-level test setup with a recorded tool-separation rate of 2 mm/min. The principal intended difference was the deposition strategy. The rule-based curved-layer component reached 3.89 kN, whereas the plotted planar-layer response indicates approximately 1.53 kN.

One component was tested per strategy, so these results provide a descriptive comparison of the two manufactured brackets. They demonstrate the implementation of the local deposition-design rules at component level. Repeatability and the separate contributions of deposition orientation and manufacturing quality remain to be established.

## 3. Discussion

The results support coordinating layer-plane orientation with filament arrangement within each layer. Compression and transverse load transfer coexist in the bolted regions, so one direction alone does not describe all local requirements. The compression benchmark informs a layer-plane target, while the hole-related benchmarks inform filament arrangements. These choices are conditional on the investigated mechanical and manufacturing conditions.

AAAAAAAA had the highest mean peak force in both hole-related benchmarks, supporting Strategy A as a starting candidate among the tested configurations. DACBBCAD changes the layer sequence and offsets reinforcement together. Its response therefore does not establish that reinforcement or weak path connections caused the lower mean peak force.

The numerical ranking agreed with the bearing comparison but reversed the order of ACACACAC and DACBBCAD in open-hole tension. The elastic Tsai–Wu model provides qualitative screening under the adopted material assumptions. Yilmaz and Eltahir [39] also reported larger strength-prediction discrepancies at intermediate raster angles than at 0° and 90° in printed PLA. Their result supports caution with off-axis prediction.

Table 6 states the material, process, geometry, and loading basis of the rules. The geometry-dependent pin-bearing responses reported for printed PLA and ABS by Khosravani et al. [40] reinforce the need to check hole and edge-distance ratios when transferring the rules to other geometries.

The robotic implementation combines path planning with robot-motion and extrusion-system development. Their coordination may affect deposited geometry and bonding, but these effects were not quantified. The present results do not establish manufacturing defects as the cause of the observed variation. Using the same robot for both component strategies provides a common platform, but different paths and robot orientations can still produce different manufacturing conditions.

Fang et al. [14] reported 3.47 kN for their curved-layer bracket, placing the present result at a similar force scale. Differences in the manufacturing platform, material grade, path-planning strategy, and testing procedure prevent attribution of the difference in peak force to the proposed rules.

The proposed method contributes a structured connection between local mechanical conditions, benchmark evidence, and deposition decisions. It complements detailed path-generation methods by providing local orientation targets and filament strategies as inputs. Translating these inputs into continuous, manufacturable paths and verifying the resulting component response remain essential parts of the workflow.

## 4. Conclusions and Outlook

This study presents a five-step method connecting functional region identification, benchmark abstraction, material characterization, comparative evaluation, and component-level transfer. Compression is evaluated experimentally, while the hole-related strategies are screened numerically and selected configurations are tested physically. The contribution is the conversion of this evidence into conditional local deposition-design rules.

For the tested hole-related cases, AAAAAAAA is the preferred starting candidate based on the sample-mean peak forces. Offset reinforcement should be assessed within the complete filament arrangement because its separate contribution was not isolated. The compression comparison supports a transverse layer-plane target within the tested conditions and manufacturing limits. The component pair demonstrates implementation of these choices. Broader applicability, quantitative failure prediction, and repeatable improvement require further validation.

Future work should prioritize replicated component tests with local displacement measurements. Matched planar components made on the robot and a commercial printer would assess combined platform and process effects. Comparing planar and curved components made on the robot would assess the implemented strategies within one platform. These comparisons would not directly predict an ideal curved-layer strength. Numerical development should address parameter sensitivity and progressive damage, supported by independent validation.

For model-based systems engineering, functional region records could be attached to CAD features and selected surfaces. Each record could specify the local mechanical condition, orientation target, path-connection requirement, material and process limits, and supporting evidence. Controls for boundary deposition direction and the number of boundary reinforcement curves could be extended from planar to curved slicing software for multi-axis FFF. Determining optimal deposition orientations for multi-axis FFF remains an active research topic. The strategies derived in this work provide guidance for selecting orientations suited to the local mechanical conditions. Their implementation in path-generation pipelines could use predefined direction fields or optimization objectives based on the stress tensor. These inputs could guide path generation toward the local deposition strategies specified by the rules.

## Figures and Tables

**Figure 1:**
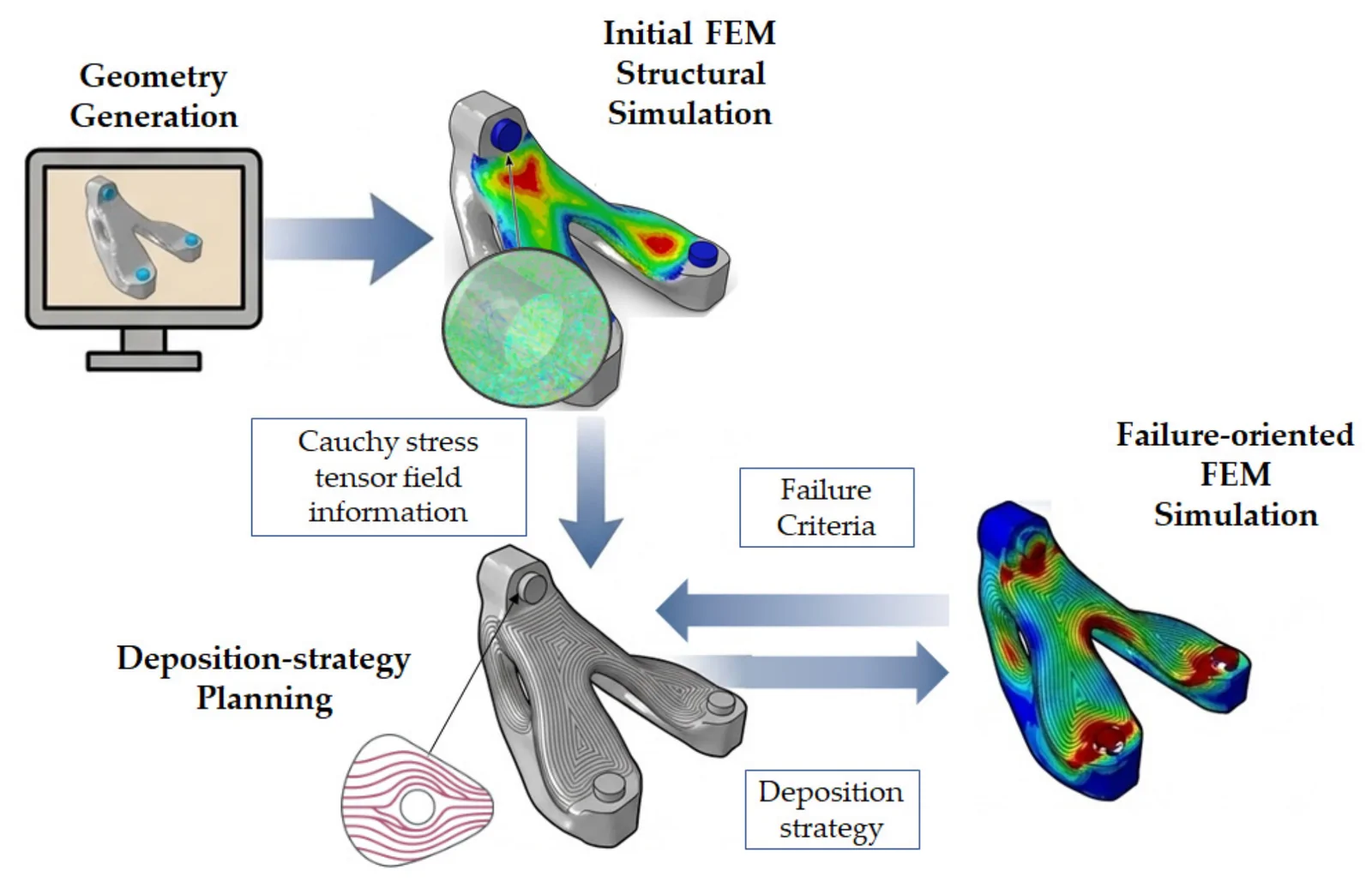
Iterative workflow linking geometry generation, initial FEM structural simulation, deposition-strategy planning, and failure-oriented FEM simulation for multi-axis FFF components [20,24].

**Figure 2:**
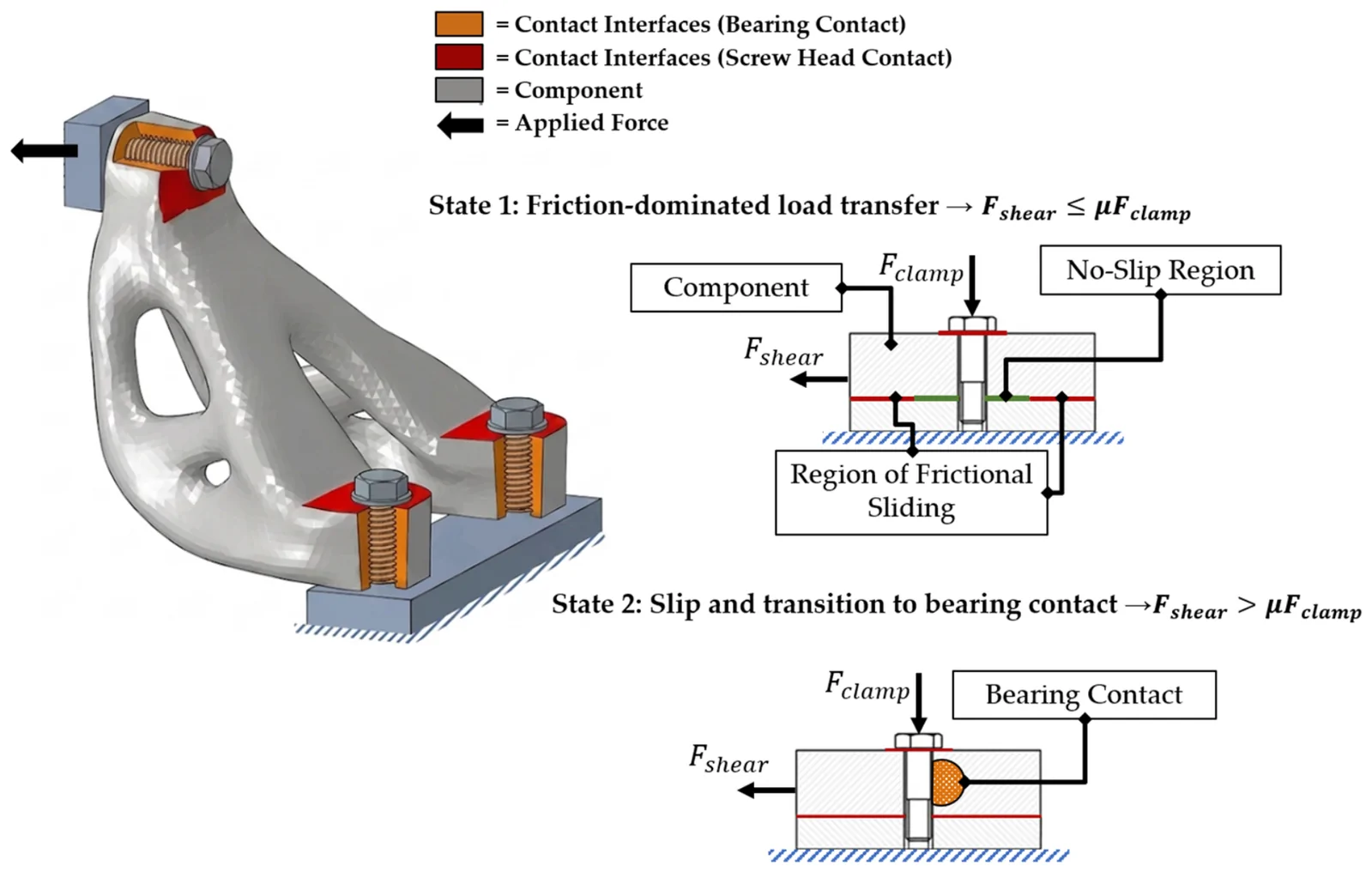
Representative load-bearing bracket with bolted-hole functional regions, contact interfaces, and load-transfer mechanisms.

**Figure 3:**
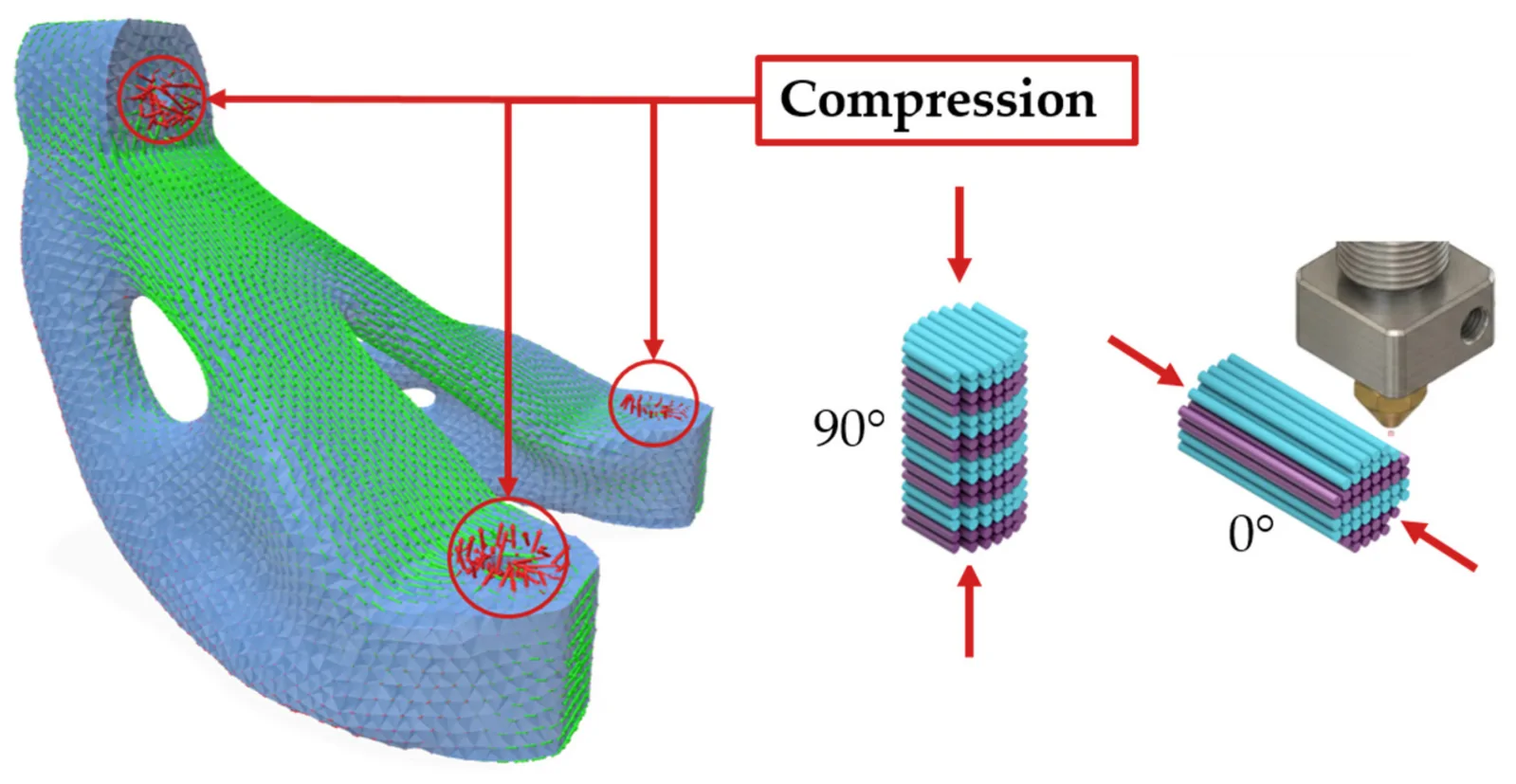
Compression-related benchmark abstraction of bolt-head contact interfaces and support reactions in the representative bracket. Red denotes compression, and green denotes tension.

**Figure 4:**
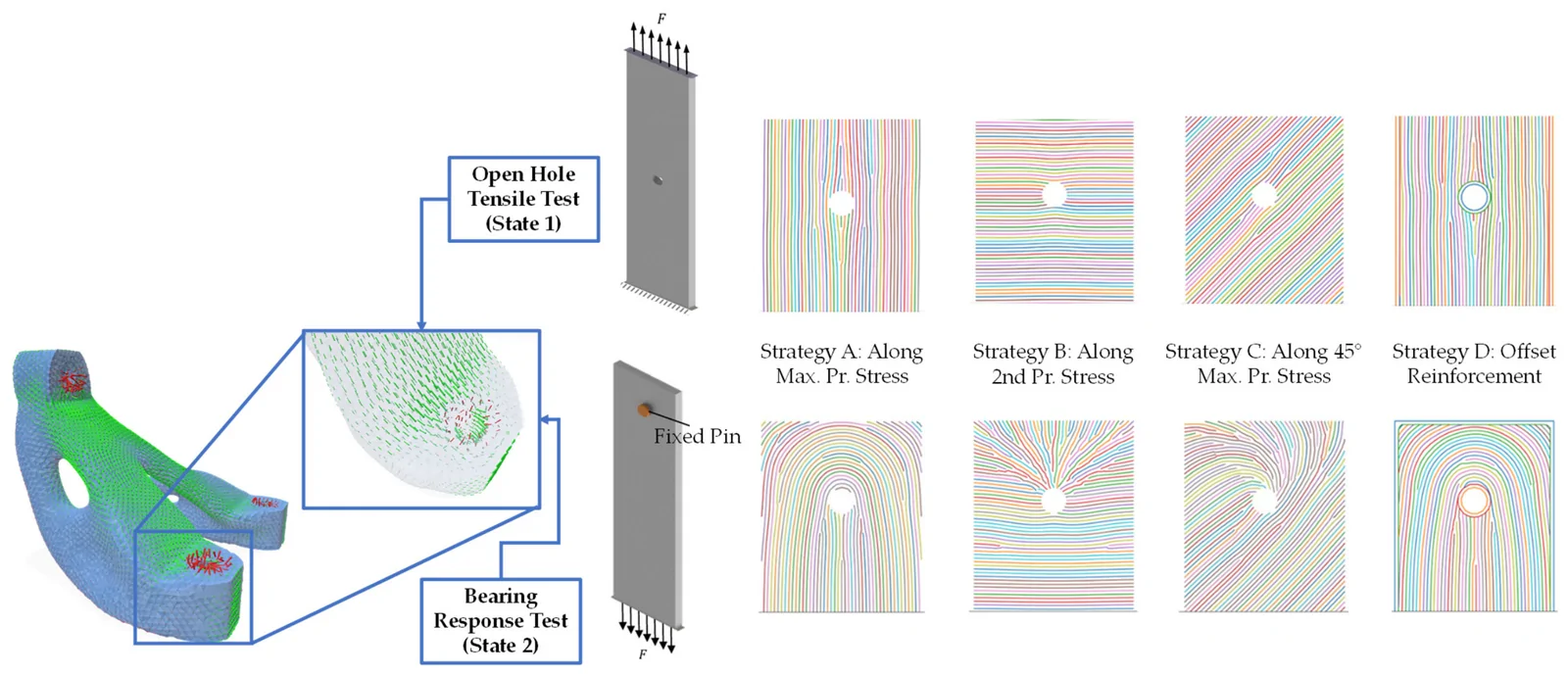
Open-hole tensile and bearing-response benchmark cases with candidate deposition strategies.

**Figure 5:**
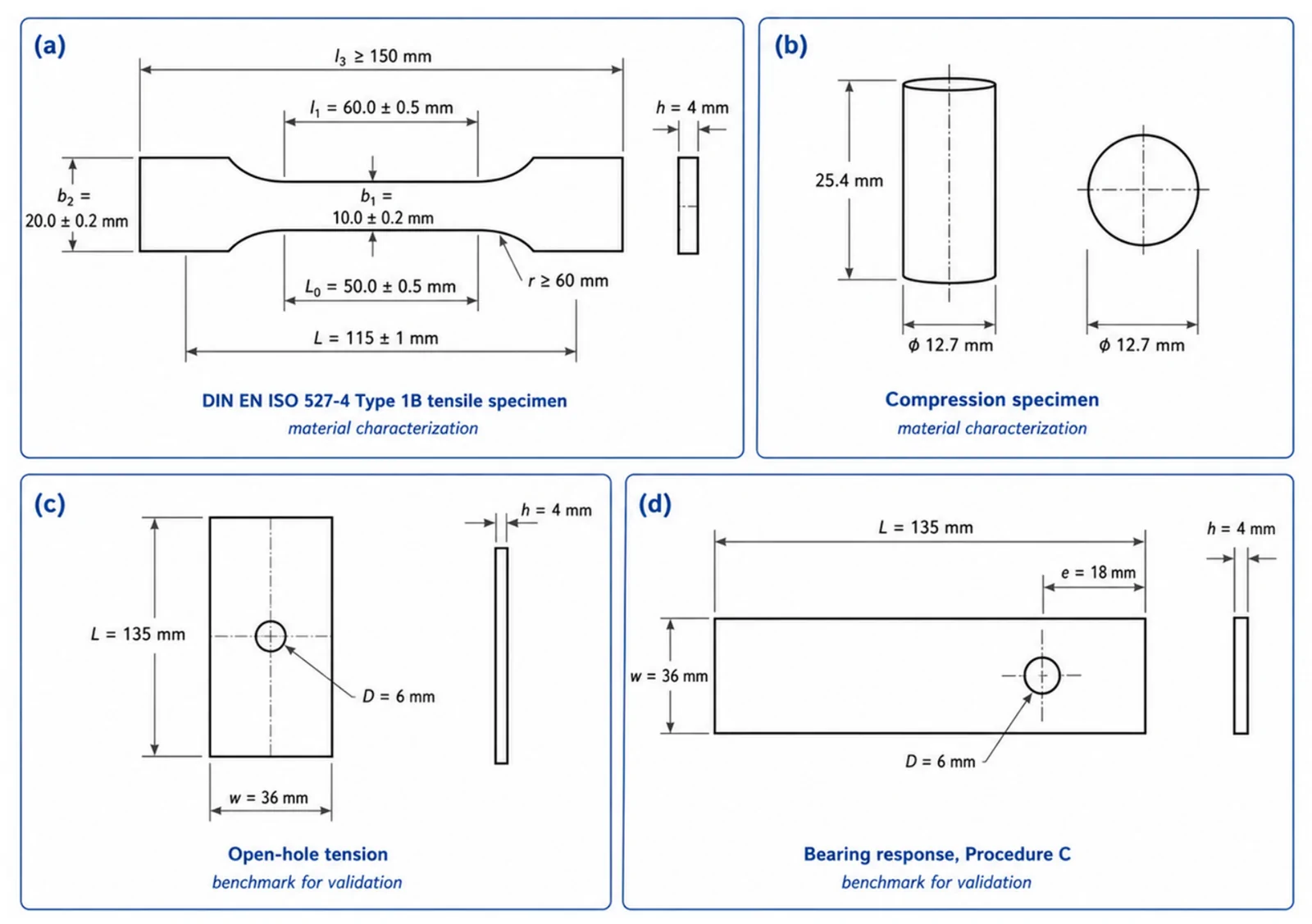
Benchmark geometries used for material characterization and functional region benchmark evaluation: (**a**) tensile test based on DIN EN ISO 527-4, Type 1B [33]; (**b**) compression test based on ASTM D695 [34]; (**c**) open-hole tensile test based on ASTM D5766/D5766M [35]; (**d**) bearing-response test based on ASTM D5961/D5961M [36] Procedure C.

**Figure 6:**
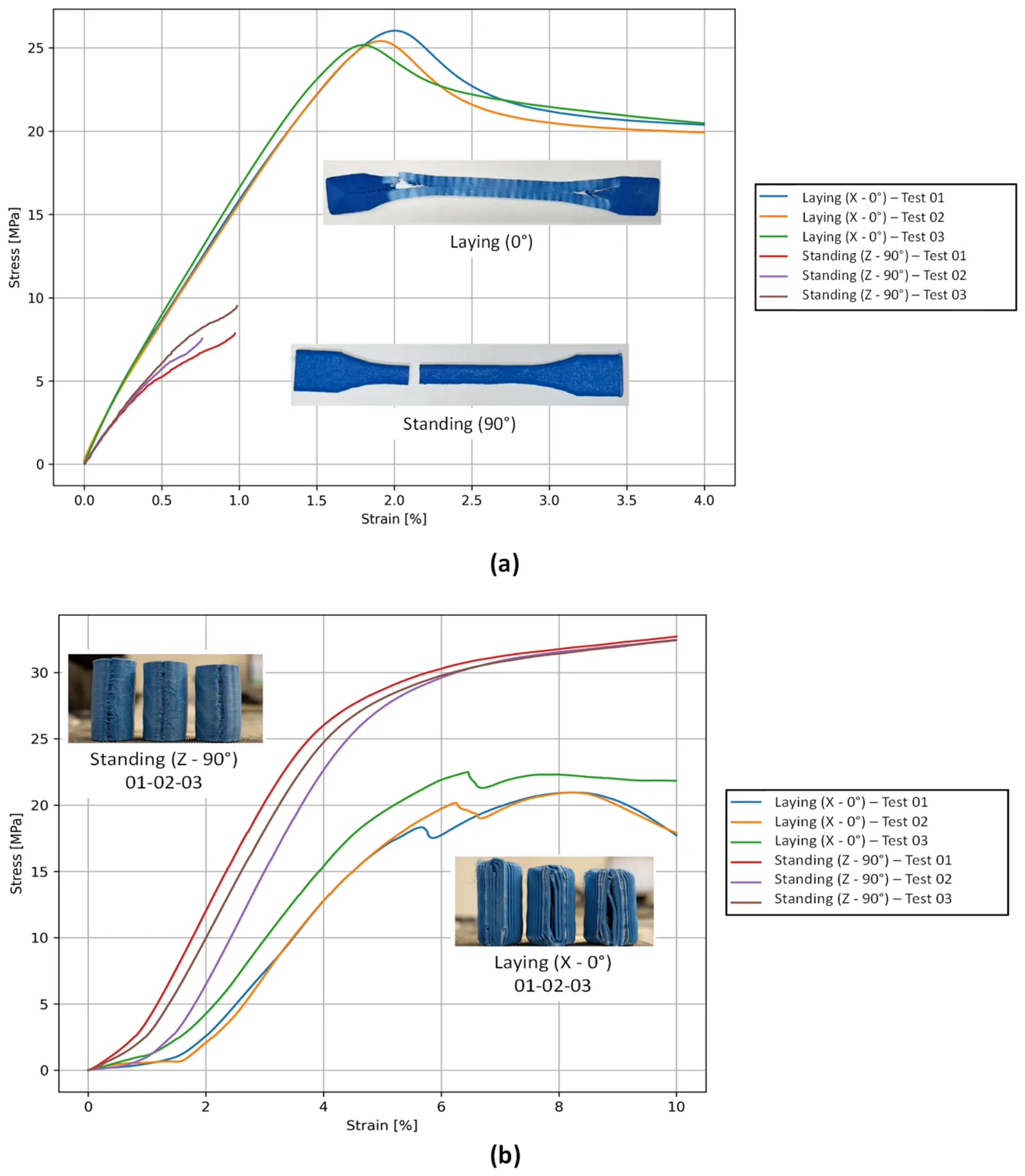
Material characterization of robot-printed PLA: (**a**) tensile stress–strain curves and representative fractured specimens for 0° and 90° filament orientations. (**b**) Compressive stress–strain curves and representative compressed specimens for 0° and 90° filament orientations.

**Figure 7:**
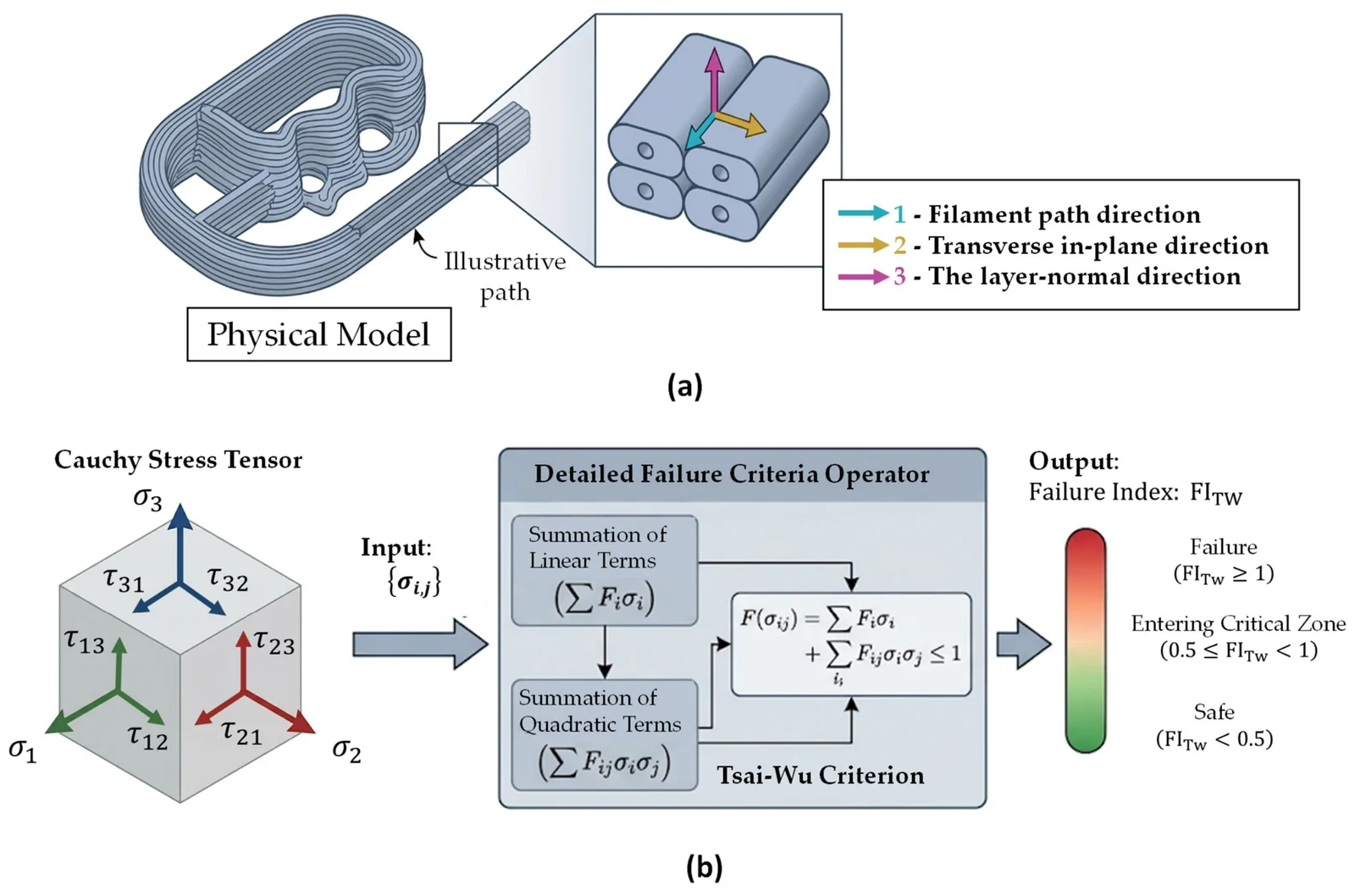
Deposition-path-driven orthotropic material assignment and failure-index calculation: (**a**) local material coordinate system; (**b**) local stress components used to compute the 3-D Tsai–Wu failure index.

**Figure 8:**
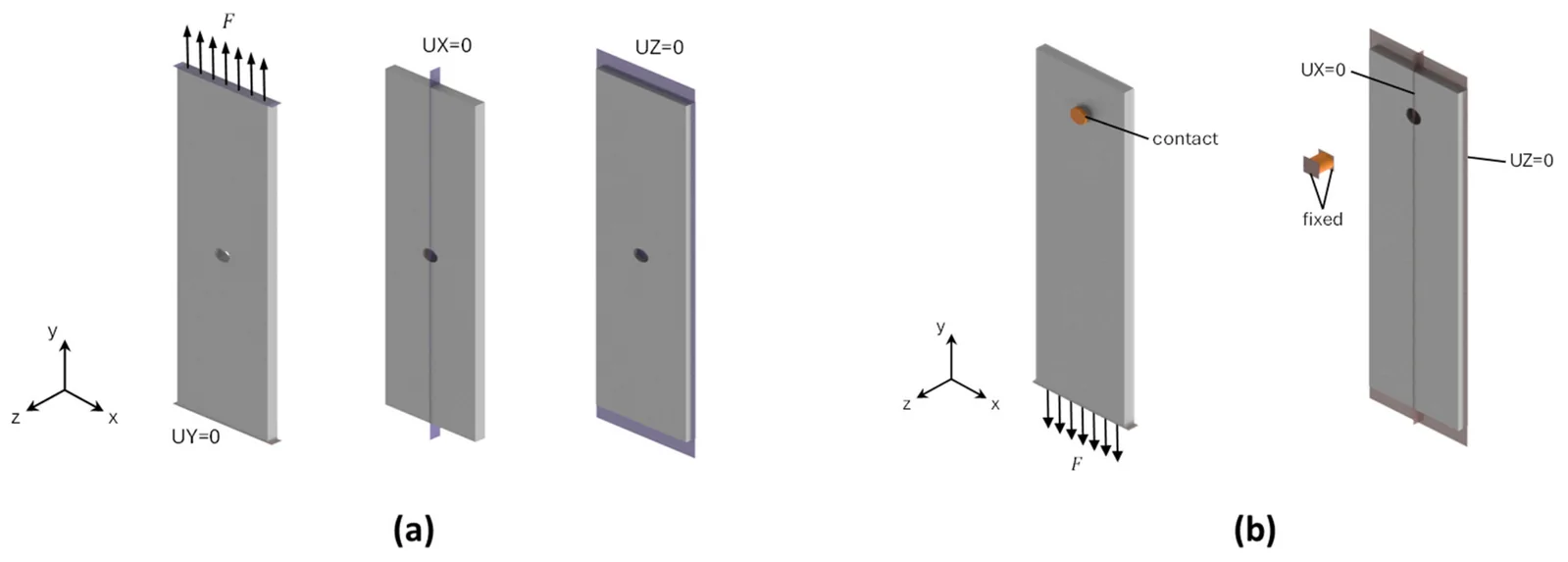
Boundary conditions and load application for (**a**) the open-hole tensile benchmark and (**b**) the bearing-response benchmark.

**Figure 9:**
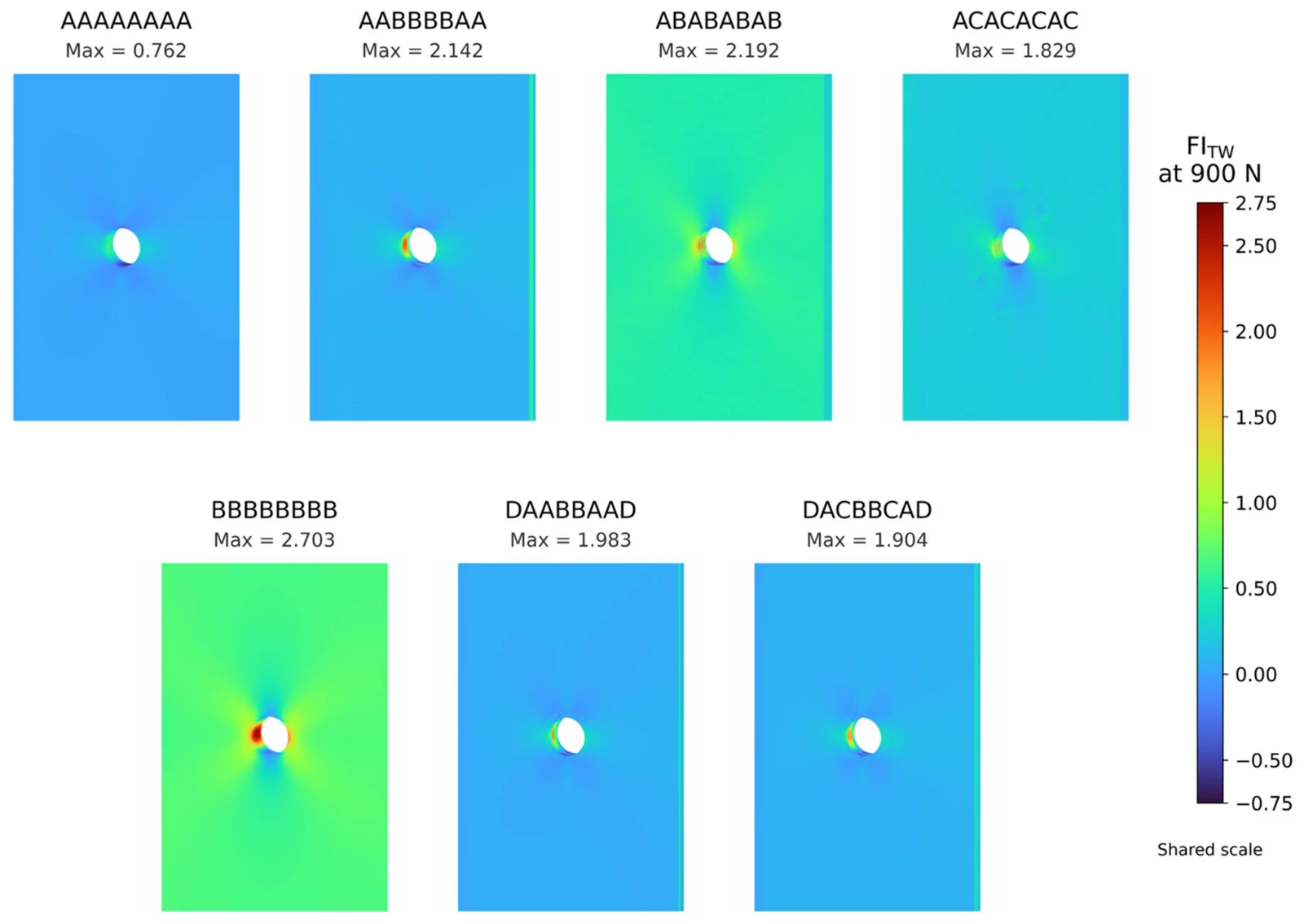
Tsai–Wu failure-initiation-index fields for the open-hole tensile stacking configurations at the common numerical screening load of 900 N.

**Figure 10:**
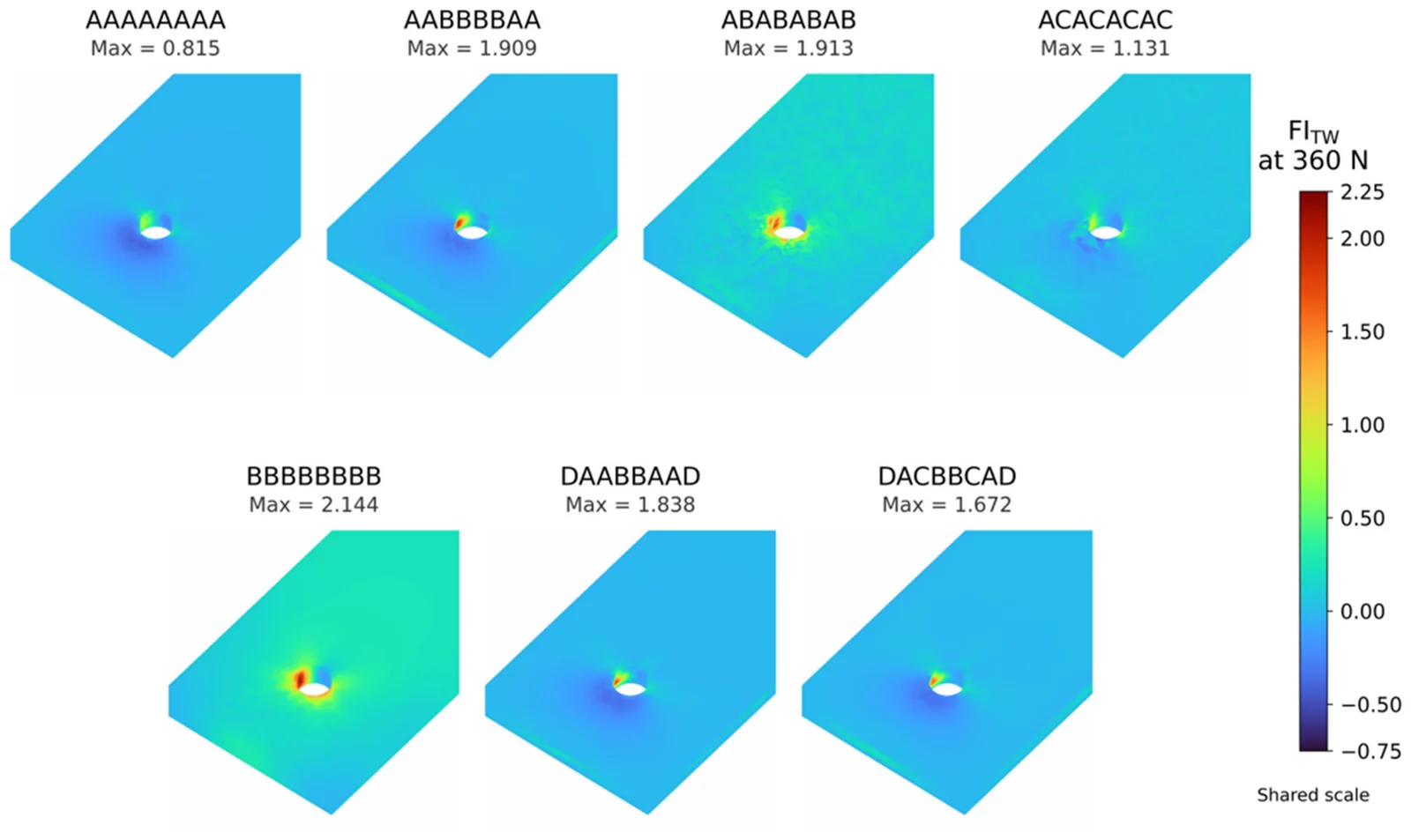
Tsai–Wu failure-initiation-index fields at the loaded hole boundary for the bearing-response stacking configurations at the common numerical screening load of 360 N.

**Figure 11:**
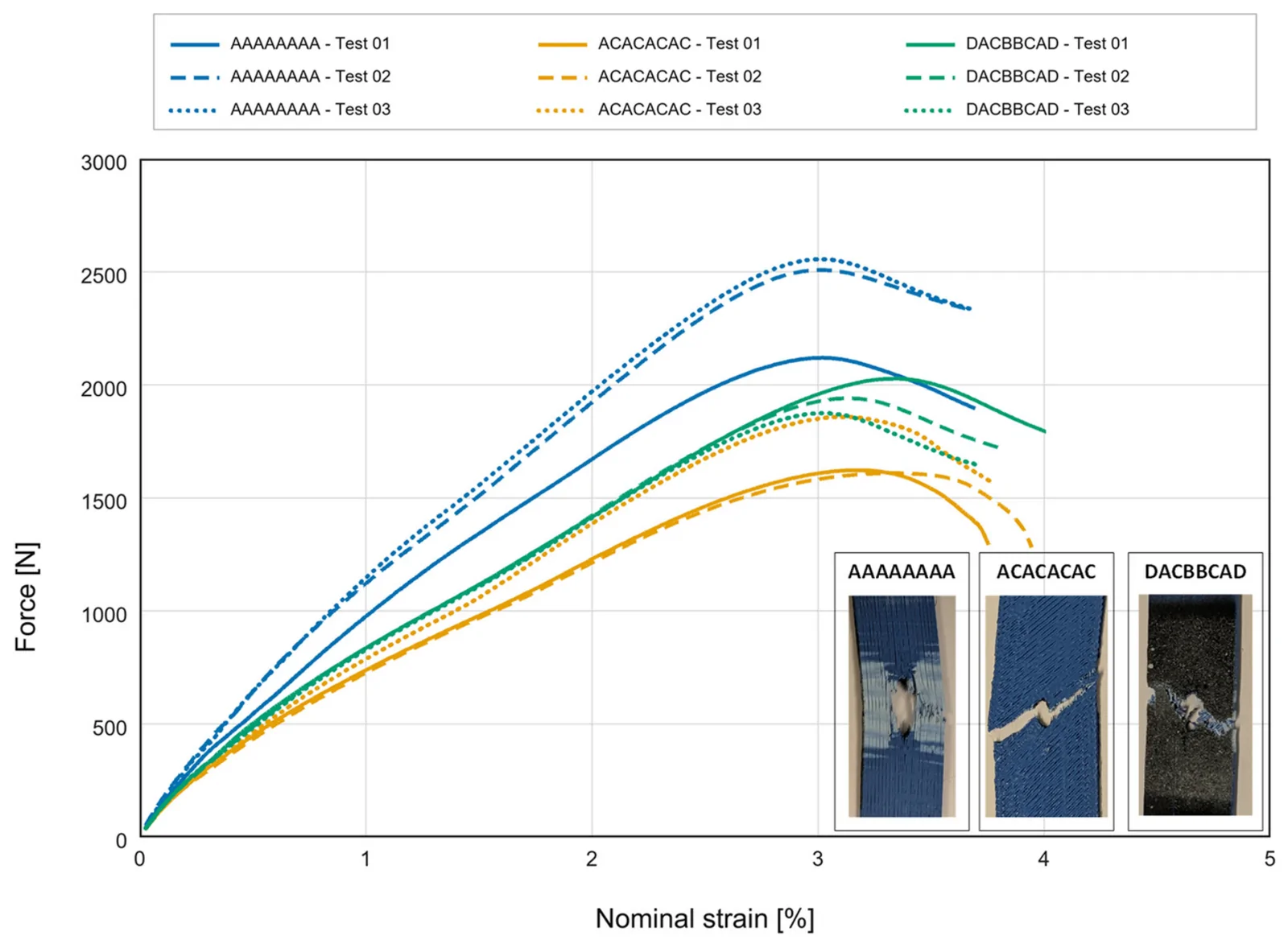
Experimental open-hole tensile response for selected stacking configurations. Force–strain curves and representative fractured specimens.

**Figure 12:**
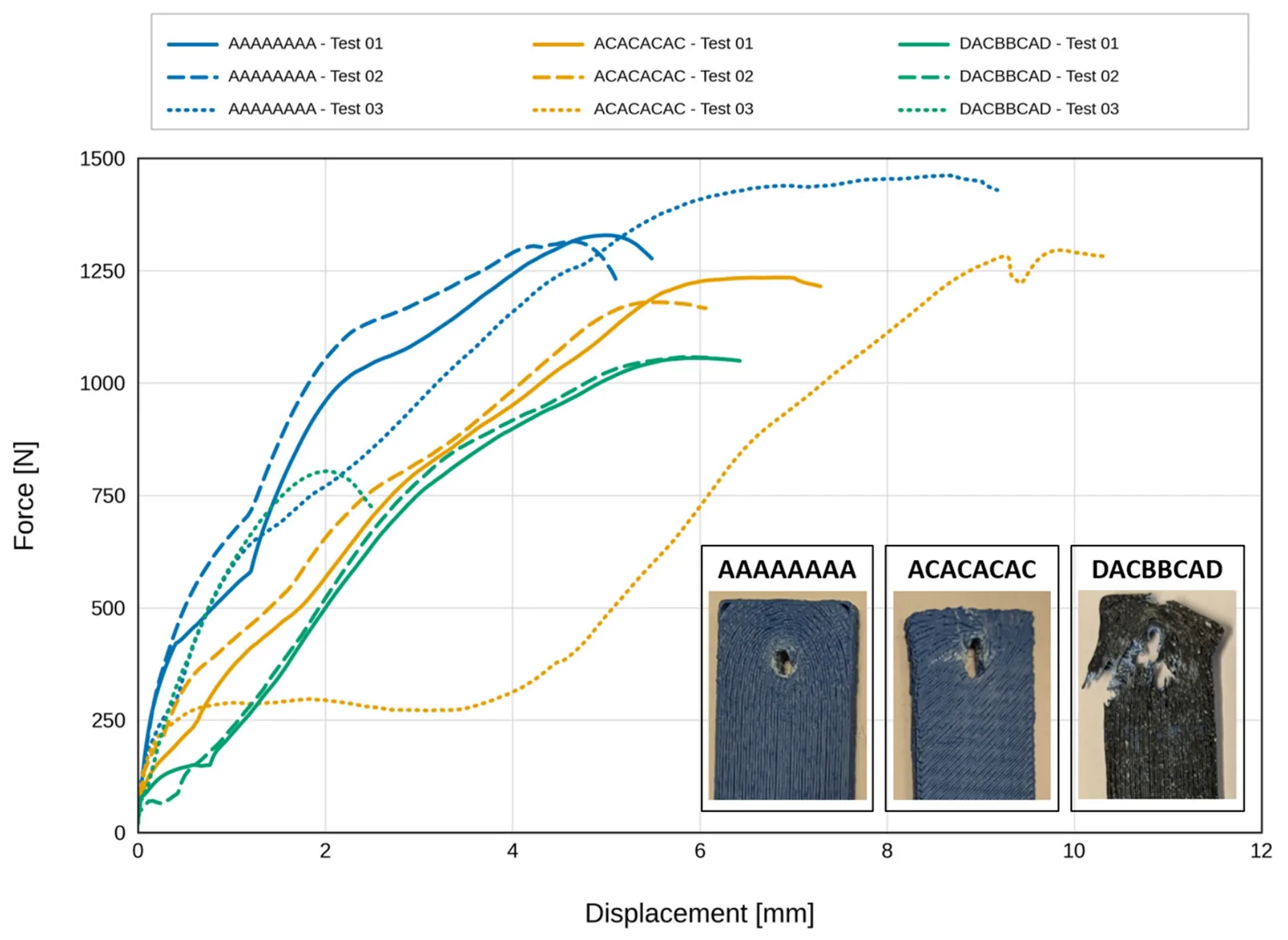
Experimental bearing-response results for selected stacking configurations. Force–displacement curves with representative failed specimens.

**Figure 13:**
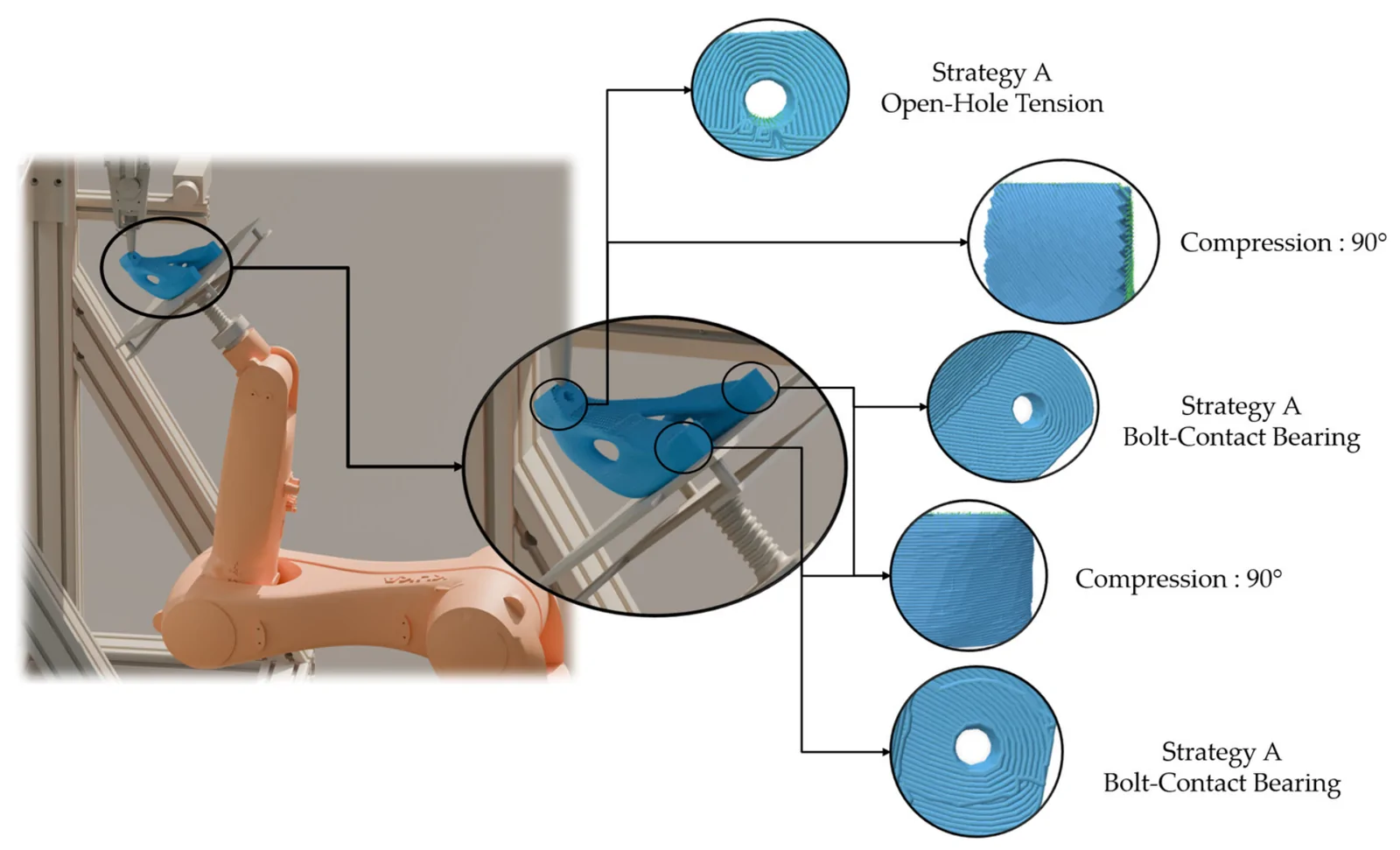
Robotic manufacturing setup and local deposition-strategy assignments for the representative bracket.

**Figure 14:**
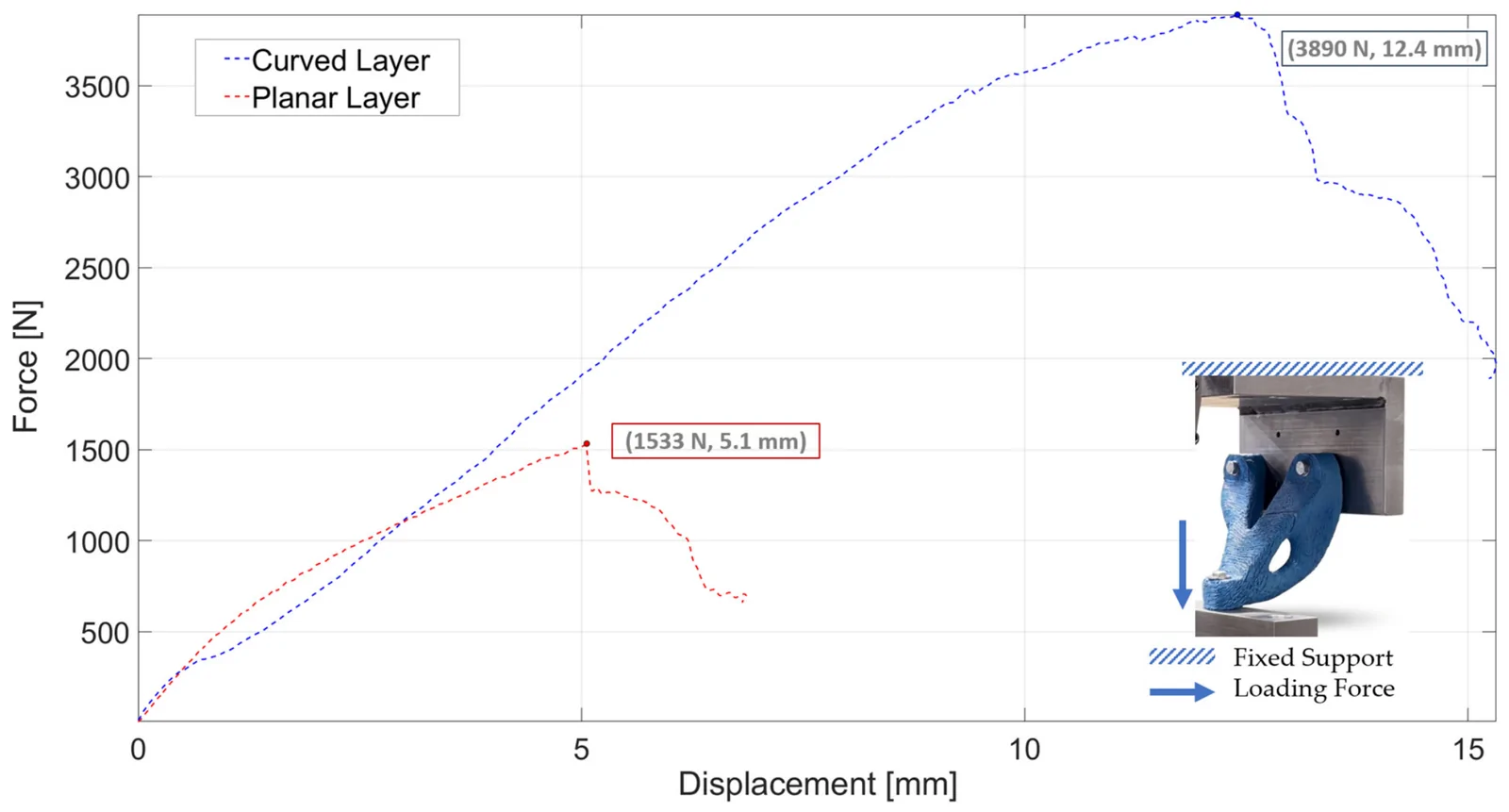
Component test setup and force–displacement responses of the planar-layer and curved-layer brackets.

**Table 1:** Stacking configurations for the open-hole tensile and bearing-response specimens and their design features.

Layer Stacking	Comparison Aspect
AAAAAAAA	Strategy A throughout all layers
BBBBBBBB	Strategy B throughout all layers
AABBBBAA	Grouped A/B layers
ABABABAB	Alternating A/B layers
ACACACAC	Alternating A/C layers incorporating the 45° strategy
DAABBAAD	A/B stacking with local offset reinforcement in the outer D layers
DACBBCAD	Combined A/B/C stacking and local offset reinforcement in the outer D layers

**Table 2:** Elastic properties used in the orthotropic material model.

Material	$E_{1}$ [MPa]	$E_{2}$ [MPa]	$E_{3}$ [MPa]	$\nu_{12}$ [-]	$\nu_{13}$ [-]	$\nu_{23}$ [-]	$G_{12}$ [MPa]	$G_{13}$ [MPa]	$G_{23}$ [MPa]
PLA-Robot	1910	1230 *	1230	0.43 *	0.37 *	0.4 *	919 *	844 *	900 *

Note: * Values marked with an asterisk were not measured for the investigated robot-printed PLA.

**Table 3:** Strength parameters used for the Tsai–Wu failure criterion.

Material	$X_{T}$ [MPa]	$X_{C}$ [MPa]	$Y_{T}$ [MPa]	$Y_{C}$ [MPa]	$Z_{T}$ [MPa]	$Z_{C}$ [MPa]	$S_{12}$ [MPa]	$S_{13}$ [MPa]	$S_{23}$ [MPa]
PLA-Robot	25.6	21.5	8.68 *	35.8 *	8.68	35.8	25.5 *	25.5 *	25.5 *

Note: * Values marked with an asterisk were not measured for the investigated robot-printed PLA. $E_{2}$, $Y_{T}$, and $Y_{C}$ were set equal to the experimental values in direction 3; $\nu_{12}$, $\nu_{13}$, $G_{12}$, $G_{13}$, $S_{12}$ were adopted from comparable PLA data reported in [37]; $\nu_{23}$, $G_{23}$ were assigned intermediate modeling values; $S_{13}$, $S_{23}$ were set equal to $S_{12}$.

**Table 4:** Maximum dimensionless Tsai–Wu failure-initiation index and critical location for each open-hole tensile stacking configuration at the common screening load of 900 N.

Configuration	Maximum Tsai–Wu Failure-Initiation Index, ${FI}_{TW}$, at 900 N [-]	Critical Location and Numerical Interpretation
AAAAAAAA	0.762	Hole boundary, localized maximum below the modeled initiation threshold
BBBBBBBB	2.703	Hole boundary, largest maximum, and broader elevated-index region
AABBBBAA	2.142	Hole boundary, grouped A/B layers
ABABABAB	2.192	Hole boundary, alternating A/B layers
ACACACAC	1.829	Hole boundary, alternating A/C layers
DAABBAAD	1.983	Hole boundary, A/B layers with local offset reinforcement
DACBBCAD	1.904	Hole boundary, A/B/C layers with local offset reinforcement

**Table 5:** Maximum dimensionless Tsai–Wu failure-initiation index and critical location for each bearing-response stacking configuration at the common screening load of 360 N.

Configuration	Maximum Tsai–Wu Failure-Initiation Index, ${FI}_{TW}$, at 360 N [-]	Critical Location and Numerical Interpretation
AAAAAAAA	0.815	Loaded hole boundary, localized maximum below the modeled initiation threshold
BBBBBBBB	2.144	Loaded hole boundary, largest maximum
AABBBBAA	1.909	Loaded hole boundary, grouped A/B layers
ABABABAB	1.913	Loaded hole boundary, alternating A/B layers
ACACACAC	1.131	Loaded hole boundary, alternating A/C layers
DAABBAAD	1.838	Loaded hole boundary, A/B layers with local offset reinforcement
DACBBCAD	1.672	Loaded hole boundary, A/B/C layers with local offset reinforcement

**Table 6:** Local deposition-design rules with documented conditions and checks before transfer. (**a**) Shared material, process, and evaluation basis. (**b**) Local deposition-design rules.

(**a**)				
**Input**	**Documented Values and Scope**			
Material	Blue PolyTerra PLA (Polymaker); 1.75 mm feedstock filament; robot-printed material designation PLA-Robot.			
Directional properties	Measured *E*_1_ = 1910 MPa; *E*_3_ = 1230 MPa; tensile strengths *X_T_* = 25.6 MPa and *Z_T_* = 8.68 MPa. Reported compression inputs: *X_C_* = 21.5 MPa; *Z_C_* = 35.8 MPa. Direction 1 follows the filament; direction 3 is normal to the layers. Full parameter set: Table 2 and Table 3.			
Deposition settings	Nozzle and nominal deposition width: 0.8 mm; layer height: 0.5 mm; nozzle temperature: 200 °C; variable deposition speed, maximum 70 mm/s. KUKA KR 6 R900 SIXX; moving platform/fixed nozzle; downhand deposition; velocity-dependent extrusion; one nozzle-directed cooling fan.			
Physical evaluation	Hole benchmarks: eight layers, nominal thickness 4 mm; three specimens per tested configuration. Axial tension for open-hole specimens; bolt contact for bearing specimens; axial compression for compression specimens. Recorded tool-separation rate: 2 mm/min.			
Numerical evaluation	Local orthotropic material assignment and elastic Tsai–Wu screening. Prescribed forces: 900 N in open-hole tension and 360 N in bearing. Compression rule: experimental evidence only. These screening forces are not allowable service loads or predicted failure loads.			
Transfer to a new component	Check material and process compatibility, local load-transfer condition, geometry, and path connections. Update material inputs or repeat the relevant benchmark evaluation when those assumptions change. Verify the resulting component response.			
(**b**)				
**Functional Region Type**	**Geometry and Local Load Transfer**	**Deposition Principles**	**Experimental Evidence**	**Application Limits**
Compression-related contact region	Cylinder: diameter 12.7 mm; height 25.4 mm. Axial compression represents local bolt-head or support contact.	Evaluate 90° layers, transverse to compression, against the tested 0° orientation. Check whether the orientation is attainable with downhand deposition.	90°: progressive compaction. 0°: mid-plane separation associated with buckling. Reported compression inputs: 35.8 versus 21.5 MPa.	Contact direction; lateral support; attainable layer orientation. The tests do not define a permissible angle interval and calibrated local compression failure law.
Hole without direct bolt–hole contact	Nominal *L* = 135, *w* = 36, *t* = 4, *D* = 6 mm. *w*/*D* = 6; *D*/*t* = 1.5. Tensile load passes around the hole.	Use AAAAAAAA as the starting candidate. Preserve connected paths around the hole and through the surrounding material. Compare mixed orientations or offsets against this candidate.	Mean peak force ± SD, kN: AAAAAAAA 2.395 ± 0.239 DACBBCAD 1.948 ± 0.077 ACACACAC 1.697 ± 0.139.	Hole geometry; load direction; path terminations and connections. This benchmark does not establish frictional load transfer in a clamped joint.
Hole with bearing contact	Nominal *L* = 135, *t* = 4, *D* = 6, *e* = 18 mm; *e*/*D* = 3. Bolt pressure loads the hole boundary.	Use AAAAAAAA as the starting candidate. Connect the loaded hole boundary to surrounding paths. Evaluate offset reinforcement within the complete orientation/stacking configuration.	Mean peak force ± SD, kN: AAAAAAAA 1.368 ± 0.081 ACACACAC 1.237 ± 0.058 DACBBCAD 0.972 ± 0.146.	Hole geometry and clearance; edge distance; contact direction; path connections; gripping. Grip slippage limits displacement interpretation.

## Data Availability

The original contributions presented in this study are included in the article. Further inquiries can be directed to the corresponding author.
